# Finite Element Analysis of Six Internal Fixations in the Treatment of Pauwels Type III Femoral Neck Fracture

**DOI:** 10.1111/os.14069

**Published:** 2024-05-15

**Authors:** Xiang Sun, Zhe Han, Dongdong Cao, Chao Han, Mengqi Xie, Xiantie Zeng, Qiang Dong

**Affiliations:** ^1^ Department of Hip Trauma Tianjin Hospital Tianjin China; ^2^ Tianjin University of Traditional Chinese Medicine Tianjin China; ^3^ Department of Foot and Ankle Surgery Tianjin Hospital Tianjin China

**Keywords:** Biomechanical study, Finite element analysis, Internal fixation, Pauwels type III femoral neck fracture

## Abstract

**Objective:**

The current investigation sought to utilize finite element analysis to replicate the biomechanical effects of different fixation methods, with the objective of establishing a theoretical framework for the optimal choice of modalities in managing Pauwels type III femoral neck fractures.

**Methods:**

The Pauwels type III fracture configuration, characterized by angles of 70°, was simulated in conjunction with six distinct internal fixation methods, including cannulated compression screw (CCS), dynamic hip screw (DHS), DHS with de‐rotational screw (DS), CCS with medial buttress plate (MBP), proximal femoral nail anti‐rotation (PFNA), and femoral neck system (FNS). These models were developed and refined using Geomagic and SolidWorks software. Subsequently, finite element analysis was conducted utilizing Ansys software, incorporating axial loading, torsional loading, yield loading and cyclic loading.

**Results:**

Under axial loading conditions, the peak stress values for internal fixation and the femur were found to be highest for CCS (454.4; 215.4 MPa) and CCS + MBP (797.2; 284.2 MPa), respectively. The corresponding maximum and minimum displacements for internal fixation were recorded as 6.65 mm for CCS and 6.44 mm for CCS + MBP. When subjected to torsional loading, the peak stress values for internal fixation were highest for CCS + MBP (153.6 MPa) and DHS + DS (72.8 MPa), while for the femur, the maximum and minimum peak stress values were observed for CCS + MBP (119.3 MPa) and FNS (17.6 MPa), respectively. Furthermore, the maximum and minimum displacements for internal fixation were measured as 0.249 mm for CCS + MBP and 0.205 mm for PFNA. Additionally, all six internal fixation models showed excellent performance in terms of yield load and fatigue life.

**Conclusion:**

CCS + MBP had the best initial mechanical stability in treatment for Pauwels type III fracture. However, the MBP was found to be more susceptible to shear stress, potentially increasing the risk of plate breakage. Furthermore, the DHS + DS exhibited superior biomechanical stability compared to CCS, DHS, and PFNA, thereby offering a more conducive environment for fracture healing. Additionally, it appeared that FNS represented a promising treatment strategy, warranting further validation in future studies.

## Graphical abstract

In this study, the three CCS + MBP showed the best biomechanical stability compared with the other five internal fixation models. The biomechanical stability of DHS + DS was second only to that of the three CCS + MBP, and both could be considered as a treatment for Pauwels III femoral neck fracture based on biomechanical stability alone. However, after considering the biomechanical stability, the stability of the internal fixation model, the difficulty of surgery and surgical trauma, FNS showed some advantages, so we believe that FNS can be considered as an effective method for treating Pauwels type III femoral neck fracture. CCS, cannulated compression screw; DHS, dynamic hip screw; DS, DHS with de‐rotational screw; FNS, femoral neck system; MBP, CCS with medial buttress plate; PFNA, proximal femoral nail anti‐rotation.

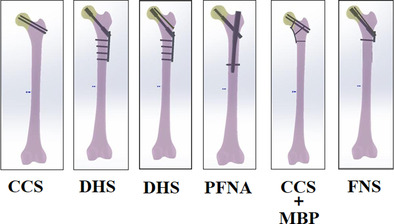

## Introduction

Femoral neck fracture is the most prevalent injury, accounting for ~60% of all hip fractures, with an estimated 2.6 million occurrences by 2025 and to 4.5 million by 2050 globally.^1^, ^2^ For elderly patients, the hip arthroplasty was regarded as mainstream treatment strategy, due to their poor bone quality, low fracture healing rate and potential bedridden complications.^3^ Conversely, for younger patients, who experienced high‐energy injuries resulting in comminuted fracture and vascular damage, taking into consideration their superior fracture healing capacity, longer life expectancy, need for enhanced hip function and the longevity of the prosthesis, contributing to various types of internal fixations, the mainstream surgical interventions were considered as the different types of internal fixations.^4^

The Pauwels classification, introduced by German physician Friedrich Pauwels in 1935, is a frequently encountered categorization of femoral neck fractures.^5^ From a biomechanical perspective, a greater Pauwels angle indicated that the fracture line is more aligned with the force of gravity, resulting in increased vertical shear stress on the fracture end.^6^ At present, the primary therapeutic goal for young individuals with Pauwels type III femoral neck fractures was to prioritize the restoration of the natural anatomical alignment of the hip joint and the provision of efficient internal fixation. This approach aimed to establish a biomechanical environment that support the healing of the fracture.^7^ Unfortunately, the occurrence of postoperative complications following the internal fixation of Pauwels type III fractures in young patients was substantial, with rates reaching 45%, Specifically, 23% of patients experienced fracture nonunion, 12% suffered from femoral head necrosis, 15% exhibited malunion, and 32% necessitated hip reconstructive surgery.^8^ Numerous evidence had confirmed that the high‐quality reduction and exceptional biomechanical stability provided by internal fixation devices were crucial factors in minimizing postoperative complications in Pauwels III type fractures.^9^ In recent times, multitudinous internal fixation devices had been developed and utilized in clinical interventions, including cannulated compression screw (CCS), dynamic hip screw (DHS), medial buttress plate (MBP), proximal femoral nail anti‐rotation (PFNA), and femoral neck system (FNS).^10^, ^11^ However, the most effective treatment approach for managing Pauwels type III femoral neck fractures remained a topic of debate.

Luttrell *et al*.^12^ investigated 272 specialists affiliated with American Academy of Orthopedic Traumatologists (OTA) on determining the preferred implant for the treatment of Pauwels type III fractures, indicating that 47% of whom favored the DHS, while 15% of whom preferred the “inverted triangle” CCS, additionally, 46% of whom acknowledged that their choice was not substantiated by existing studies, and most clinicians believed that biomechanical stability as the primary factor influencing their choice of internal fixation method. Despite increasing experimental and clinical studies attempt to determine the optimal mechanical stability of implants for treatment of Pauwels type III fracture, there was a lack of comprehensive studies that systematically compared various types of internal fixations currently utilized in clinical practice.^10^ In contrast to conventional mechanics experiments, finite element analysis was able to offer a more reliable and reproducible means of assessing biomechanical properties within the same mechanical environment, particularly when comparing various installations.^13^

Therefore, the aims of present study as follows: (i) to systematically identify the mechanical stability of six internal fixations commonly utilized in clinical practice for Pauwels type III femoral neck fracture through finite element analysis; and (ii) to determine the optimal fixation selection for treating Pauwels type III fracture in patients.

## Materials and Methods

### Development of the Normal Femur Model

Femur computed tomography (CT) data were acquired from a healthy 30‐year‐old Chinese male volunteer, weighing 70 kg, with no prior femoral or hip trauma history, using spiral CT scans with a layer thickness of 0.625 mm (Geomagic Inc., Raliegh, NC, USA), saved in DICOM format. Subsequently, Mimics 20.0 software (Materialise, Leuven, Belgium) was utilized for the three‐dimensional (3D) reconstruction of the CT images, and Geomagic Studio 2014 software (Geomagic Inc.) was employed for the optimization of the femur 3D model.

### Building the Pauwels III Femoral Neck Fracture and Internal Fixation Models

**FIGURE 1:**
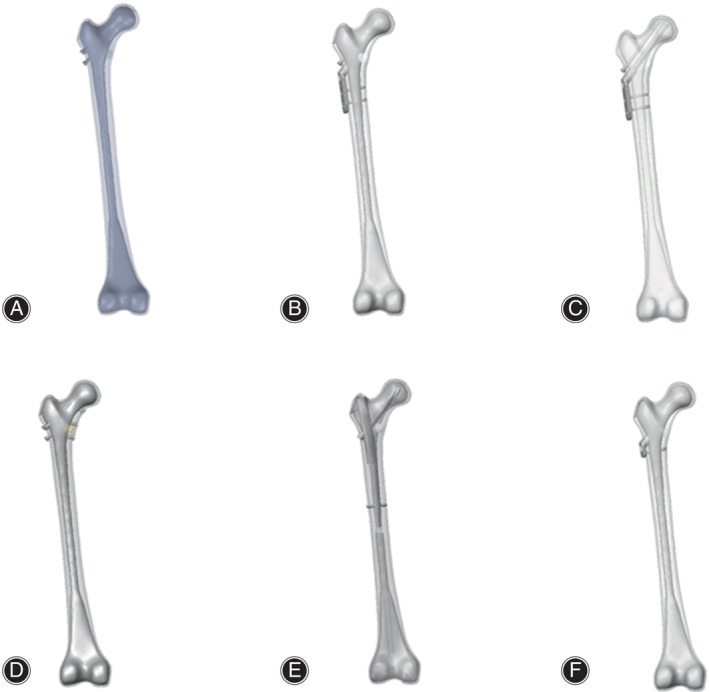
Schematic diagrams of the six internal fixation models. (A) CCS; (B) DHS; (C) DHS + DS; (D) CCS + MBP; (E) PFNA; (F) FNS. CCS, cannulated compression screw; DHS, dynamic hip screw; DS, DHS with de‐rotational screw; FNS, femoral neck system; MBP, CCS with medial buttress plate; PFNA, proximal femoral nail anti‐rotation.

Using the SolidWorks 2020 software (SolidWorks Co., Concord, MA, USA), we simulated Pauwels type III femoral neck fractures with angles of 70 ° in accordance with Pauwels' classification.^14^ According to parameters of internal fixation provided by different manufacturers and surgical methods, six type of internal fixation models were created and integrated with the Pauwels type III fracture model. The internal fixation models for fractures are detailed as follows (Figure 1).

#### CCS Model

Three screws were arranged parallel in an “inverted triangle” pattern in the proximal femur: the first screw should be inserted below the level of the lesser trochanter, near the calar, and positioned postero‐inferiorly in the femoral neck. The second and third screws should be positioned antero‐superiorly and postero‐superiorly in the femoral neck, near the femoral neck cortex. The tips of the three screws were less than 5 mm from the femoral head cortex. The diameter of the distal thread was 7.3 mm, the length of the thread was 16 mm, the screw hollow was 2.5 mm and the length of three screws were 100, 90 and 90 mm, respectively.

#### DHS Model

A two holes DHS (135° nail plate angle) was selected. The lag screw was positioned in the central and lower region of the neck of the femur, ensuring that the tip was within 5 mm of the cortex of the femoral head. Additionally, the plate was affixed to the proximal lateral cortex of the femur using two locking screws that penetrated the opposite cortex. DHS parameters including: the length of plate was 54 mm; sleeve length was 25 mm; the distal threaded of lag screw was 12 mm and length of lag screw was 100 mm; and the length of upper and lower locking screws in plate were 45 and 40 mm, respectively.

#### DHS + De‐rotational Screws (DSs) Model

A DS screw was placed parallel to the femoral neck above the lag screw in the DHS model, with the tip less than 5 mm from the femoral head cortex. The diameter of the distal thread of the DS was 7.3 mm, the length of the thread was 16 mm, the screw hollow was 2.5 mm and the length of DS is 90 mm.

#### CCS + MBP Model

According to the CCS model, a tubular plate with a slight posterior curve and four holes was positioned directly beneath the femoral neck, and three locking screws were inserted from the proximal to the distal end of the fracture site. The length, thickness and width of the MBP were 57, 1.7 and 10 mm respectively. The diameter of the locking screws was 3.5 mm and the length of locking screws were 25, 30 and 30 mm respectively.

#### PFNA

The distal portion of the intramedullary nail extended to the midsection of the femoral shaft, and the anti‐rotation blade was positioned in the lower middle portion of the femoral neck, with its tip less than 5 mm from the cortex of the femoral head. The distal end of the intramedullary nail was secured with a cortical screw that penetrated the double‐layered femoral cortex. The length and diameter of the intramedullary nail was 200 and 10 mm. The proximal anti‐rotation blade was 10 mm in diameter and 95 mm in length. The distal screw was 4.9 mm in diameter and 30 mm in length.

#### FNS

The FNS device was composed of three parts: locking plate, bolt, and anti‐rotation screw. At the proximal end of the FNS, a bolt was placed at an angle of 130° to the locking plate and an anti‐rotational screw was placed at an angle of 7.5° to the bolt. The bolt was 10 mm in diameter and 90 mm in length, and the anti‐rotation screw was 6.4 mm in diameter and 90 mm in length. At the distal end of the FNS, a 5 mm locking screw was placed to connect the locking plate to the femoral shaft.

### Mesh Generation and Material Parameters

The Hypermesh 13.0 software (Altair Engineering, Troy, MI, USA) was employed for polishing and meshing the 3D experimental models into 1‐mm equal‐sized facets.^15^ The number of nodes and elements of the six finite element models were shown in Table 1. Based on previous relevant studies,^16^, ^17^ the 3D models in this experiment were all assumed to be continuous, isotropic and uniform linear elastic materials. The material parameters of the bones and implants in our study were shown in Table 2.

**TABLE 1:** Number of units and nodes of six finite element models.

Models	Number of units	Number of nodes
CCS	179,772	270,603
DHS	200,102	301,083
DHS + DS	203,823	310,337
CCS + MBP	186,298	282,966
PFNA	186,001	278,233
FNS	185,801	274,054

Abbreviations: CCS, cannulated compression screw; DHS, dynamic hip screw; DS, DHS with de‐rotational screw; FNS, femoral neck system; MBP, CCS with medial buttress plate; PFNA, proximal femoral nail anti‐rotation.

**TABLE 2:** Material properties of femur and internal fixation.

Materials	Elasticity modulus (MPa)	Poisson ratio	Ultimate tensile strength (MPa)	Yield strength (MPa)	Fatigue resistance (MPa)	Elongation at break
Femoral cortical bone	16,800	0.30	106	157	‐	‐
Femoral cancellous bone	840	0.29	‐	5.4	450	12%
Titanium alloy (Ti‐6Al‐7Nb)	110,000	0.30	950	880	510	10%

### Contact Surface Settings

According to previous studies,^14^, ^18^ it was assumed that the fracture surface of the femoral neck was completely broken and not displaced. The fracture surface was considered as a friction contact with a friction coefficient of 0.46, as well as the internal fixation devices and bone with a friction coefficient of 0.30.

### Boundary Conditions and Loading Force

**FIGURE 2:**
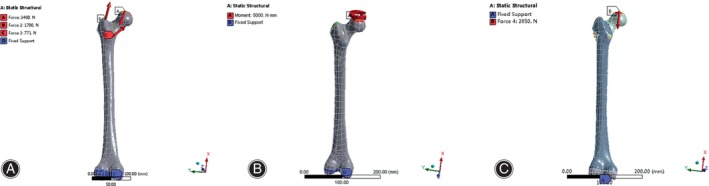
Boundaries and constraints of the finite element models. (A) Schematic diagram of axial compression loading; (B) schematic diagram of horizontal torsion loading; (C) schematic diagram of axial destructive loading.

In line with prior studies,^19^, ^20^ the axis loading and muscle forces of the hip were simplified as a pelvic force (A), an adductor force (B) and force of lateral femoral muscle (C). In this investigation, we modeled the force applied to the hip joint of an adult with a body weight of 70 kg (*G* = 700 N) during slow walking (*A* = 2.35 *G*), with a line of force at an angle of 24.5° from the femoral shaft; *B* = 1.546 G, with line of force at an angle of 29.5° from the femoral shaft; and *C* = 0.25 *G*, with line of force was parallel to the femoral shaft. Further, we subjected the femoral head to a torsional load of 5 N m, replicating the force experienced during the twisting movement of a leg lift, as described in a previous study, which simulated the force on the femoral head during the twisting movement of the leg lift.^21^ To simulate the axial compression failure test, a continuous and dynamic vertical force was applied to the femoral head, gradually increasing from 0 N until the models reached the yield strength of the materials.^21^ In addition, a vertical cyclic force of 2850 N was exerted at a frequency of 3 Hz on the femoral head, replicating the axial compression fatigue test without a specified limit on the number of cycles.^16^, ^21^, ^22^ For calculation purposes, it was assumed that the distal end of the femur was completely fixed^23^ (Figure 2). Finally, finite element analysis (FEA) Ansys 17.0 software (ANSYS, Inc., Pittsburgh, PA, USA) was applied to analyze the mechanical loading.

### Main Outcome Measures

In the context of finite element analysis, this study observed the variation in each parameter as below: (i) the displacement distribution and maximum displacement of the femur and internal fixation under varying loads; (ii) the distribution of von Mises stress and peak stress of the femur and internal fixation under different loading conditions; (iii) the yield load of the internal fixation; and (iv) the number of cyclic loading cycles required for the internal fixation to reach yield. All simulation analyses were conducted by two independent researchers at distinct time intervals.

## Results

### Stress Distribution and Peak Value of Internal Fixation

**FIGURE 3:**
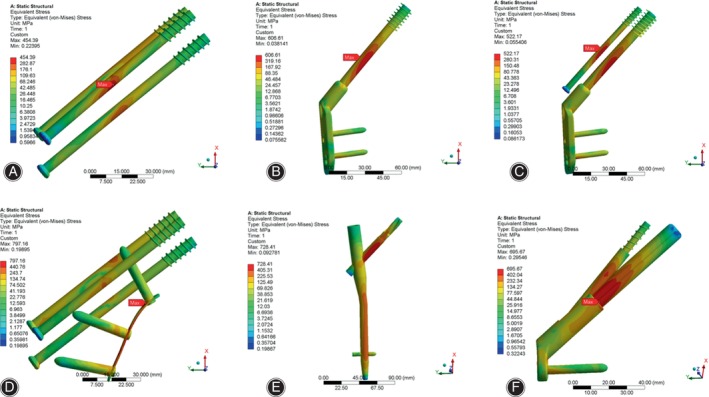
Stress distribution in internal fixation of finite element models under axial loading. (A) CCS; (B) DHS; (C) DHS + DS; (D) CCS + MBP; (E) PFNA; (F) FNS. The maximum stress distribution of internal fixations is mainly distributed on the broken end of the fracture (Red arrow). CCS, cannulated compression screw; DHS, dynamic hip screw; DS, DHS with de‐rotational screw; FNS, femoral neck system; MBP, CCS with medial buttress plate; PFNA, proximal femoral nail anti‐rotation.

As shown in Figure 3, the primary area of stress concentration for the six implant models was observed to be in the central region of the internal fixation device, near the fracture line. Also, in CCS + MBP, the stress was found to be concentrated around the screw holes on the steel plate, particularly the first screw hole. Moreover, stress was also observed to be concentrated from the middle of the nail to the upper end of the distal lock screw in the PFNA. The peak value of internal fixation stress in CCS, DHS, DHS + DS, CCS + MBP, PFNA and FNS were 454.4, 606.6, 522.2, 797.2, 728.4 and 695.7 MPa, respectively (Table 3, Figure S1). The peak stress of internal fixation in the CCS + MBP was highest, and the peak stress of the CCS was lowest.

**TABLE 3:** Peak femoral and internal fixation stresses in six internal fixation models under different loadings.

Models	Axial compression load (MPa)		Horizontal torsional load (MPa)	
	Femur	Internal fixation	Femur	Internal fixation
CCS	215.4	454.4	81.2	122.9
DHS	275.3	606.6	72.8	98.1
DHS + DS	273.8	522.2	74.3	72.8
CCS + MBP	284.2	797.2	119.3	153.6
PFNA	276.6	728.4	43.0	76.8
FNS	272.4	695.7	17.6	81.1

Abbreviations: CCS, cannulated compression screw; DHS, dynamic hip screw; DS, DHS with de‐rotational screw; FNS, femoral neck system; MBP, CCS with medial buttress plate; PFNA, proximal femoral nail anti‐rotation.

**FIGURE 4:**
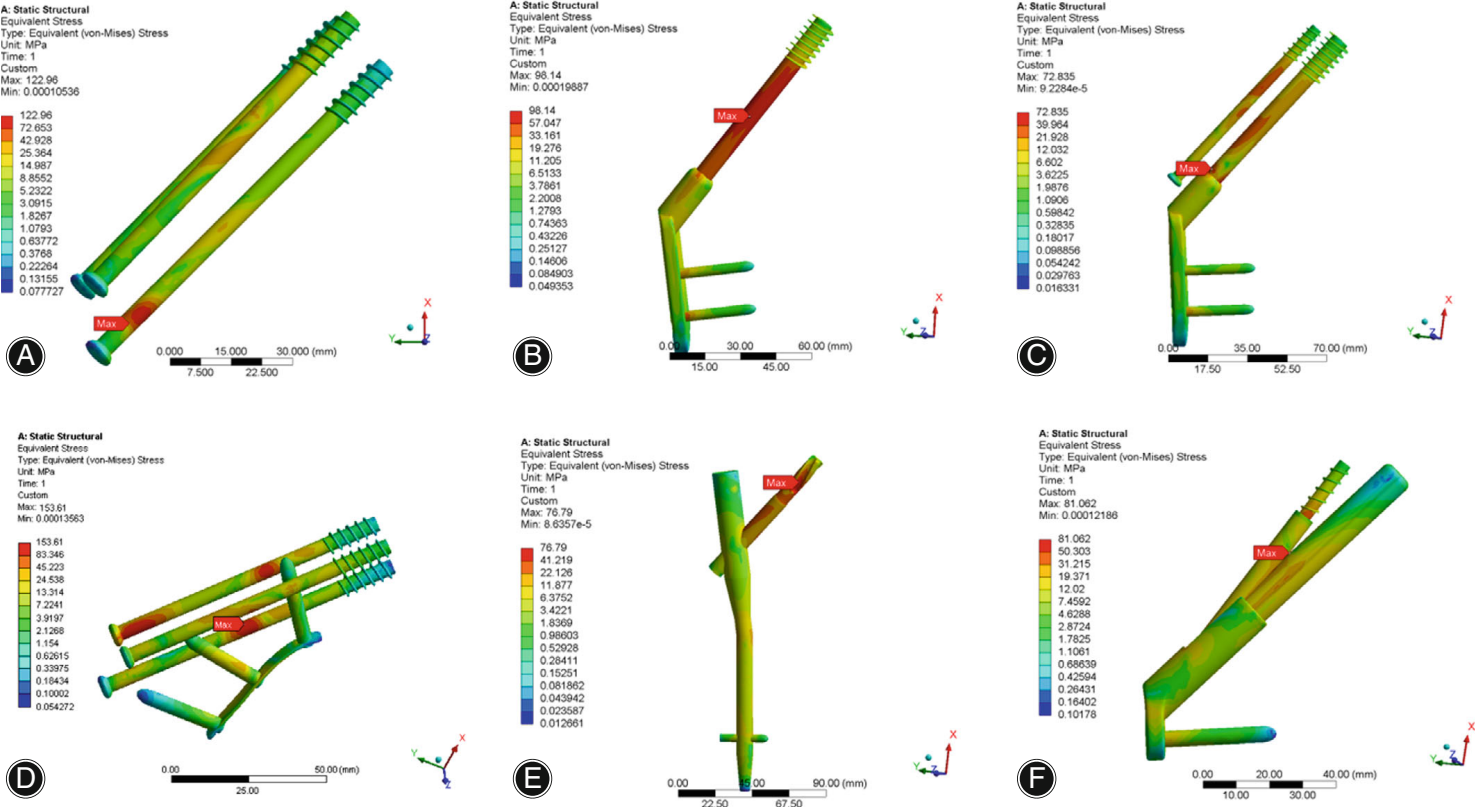
Stress distribution in internal fixation of finite element models under torsional loading. (A) CCS; (B) DHS; (C) DHS + DS; (D) CCS + MBP; (E) PFNA; (F) FNS. The maximum stress distribution of internal fixations is mainly distributed on the broken end of the fracture (Red arrow). CCS, cannulated compression screw; DHS, dynamic hip screw; DS, DHS with de‐rotational screw; FNS, femoral neck system; MBP, CCS with medial buttress plate; PFNA, proximal femoral nail anti‐rotation.

When subjected to torsional loading, the stress experienced by the six internal fixation models was focused at the interface between the internal fixation device and the fracture line. Peculiarly, in the CCS model, the stress was also concentrated at the end of the inferolateral screw (Figure 4). The peak value of internal fixation stress in CCS, DHS, DHS + DS, CCS + MBP, PFNA and FNS were 122.9,98.1, 72.8, 153.6, 76.8 and 81.1 MPa, respectively (Table 3, Figure S1). The maximal peak stress of internal fixation was CCS + MBP, and the PFNA had minimum peak stress.

### Stress Distribution and Peak Value of Femur

**FIGURE 5:**
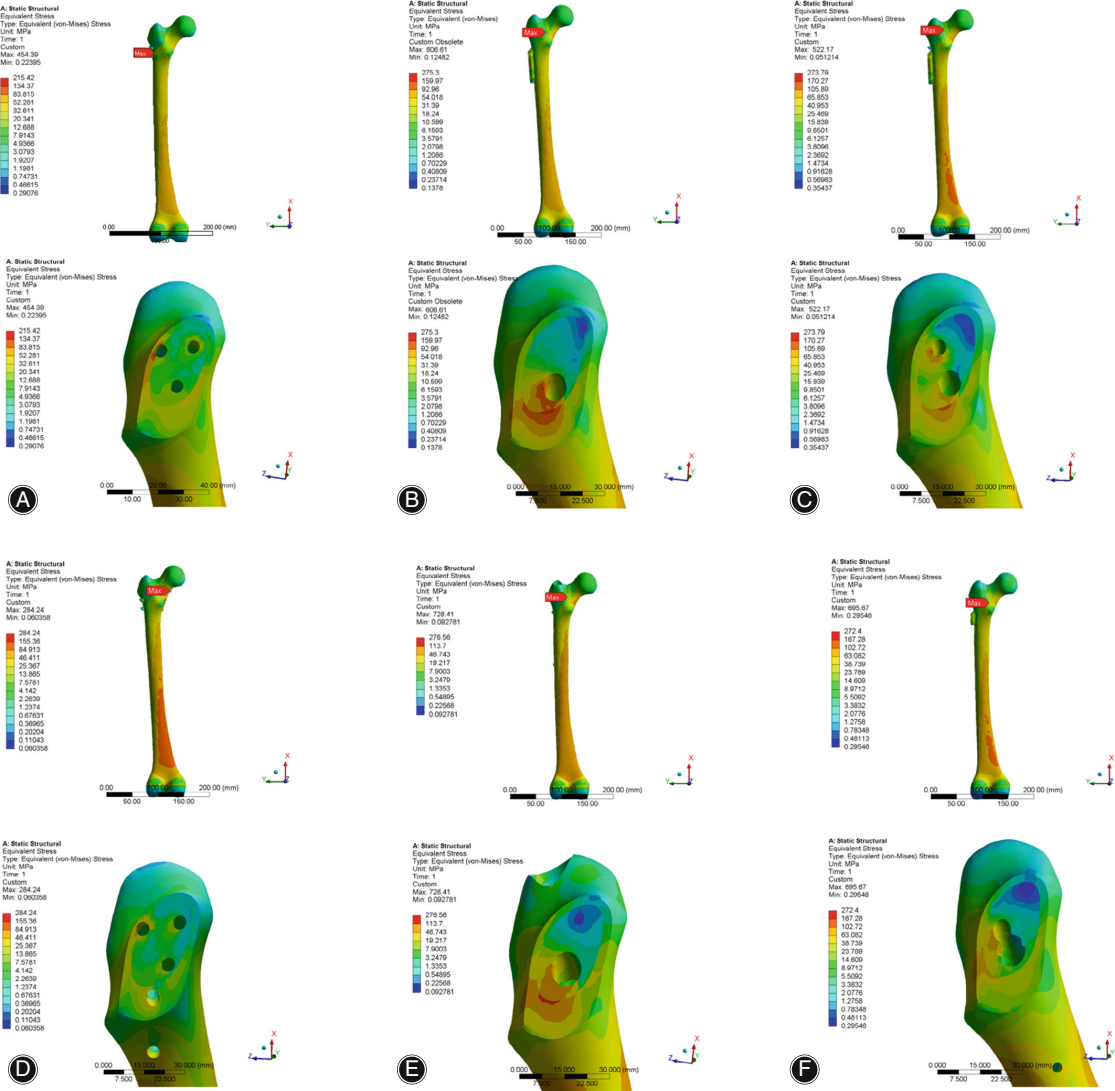
Stress distribution of femur and femur section under axial loading. (A) CCS; (B) DHS; (C) DHS + DS; (D) CCS + MBP; (E) PFNA; (F) FNS. The maximum stress distribution of femur is mainly distributed on the broken end of the fracture and femoral calcar. CCS, cannulated compression screw; DHS, dynamic hip screw; DS, DHS with de‐rotational screw; FNS, femoral neck system; MBP, CCS with medial buttress plate; PFNA, proximal femoral nail anti‐rotation.

As displayed in Figure 5, the distribution of stress in the femur mostly localized at the anterior and inferior fracture surfaces corresponding to femoral callus. Furthermore, the stress was also observed to be concentrated around the screw hole of the femoral neck in the CCS + MBP, and in the middle and lateral regions of the femoral shaft in the PFNA. The peak value of femur stress in CCS model, DHS model, DHS + DS, CCS + MBP, PFNA and FNS were 215.4, 275.3, 273.8, 284.2, 276.6, and 272.4 MPa, respectively (Table 3, Figure S1). Similarly, the peak stress of femur in the CCS + MBP was highest, and the peak stress of the CCS was lowest.

**FIGURE 6:**
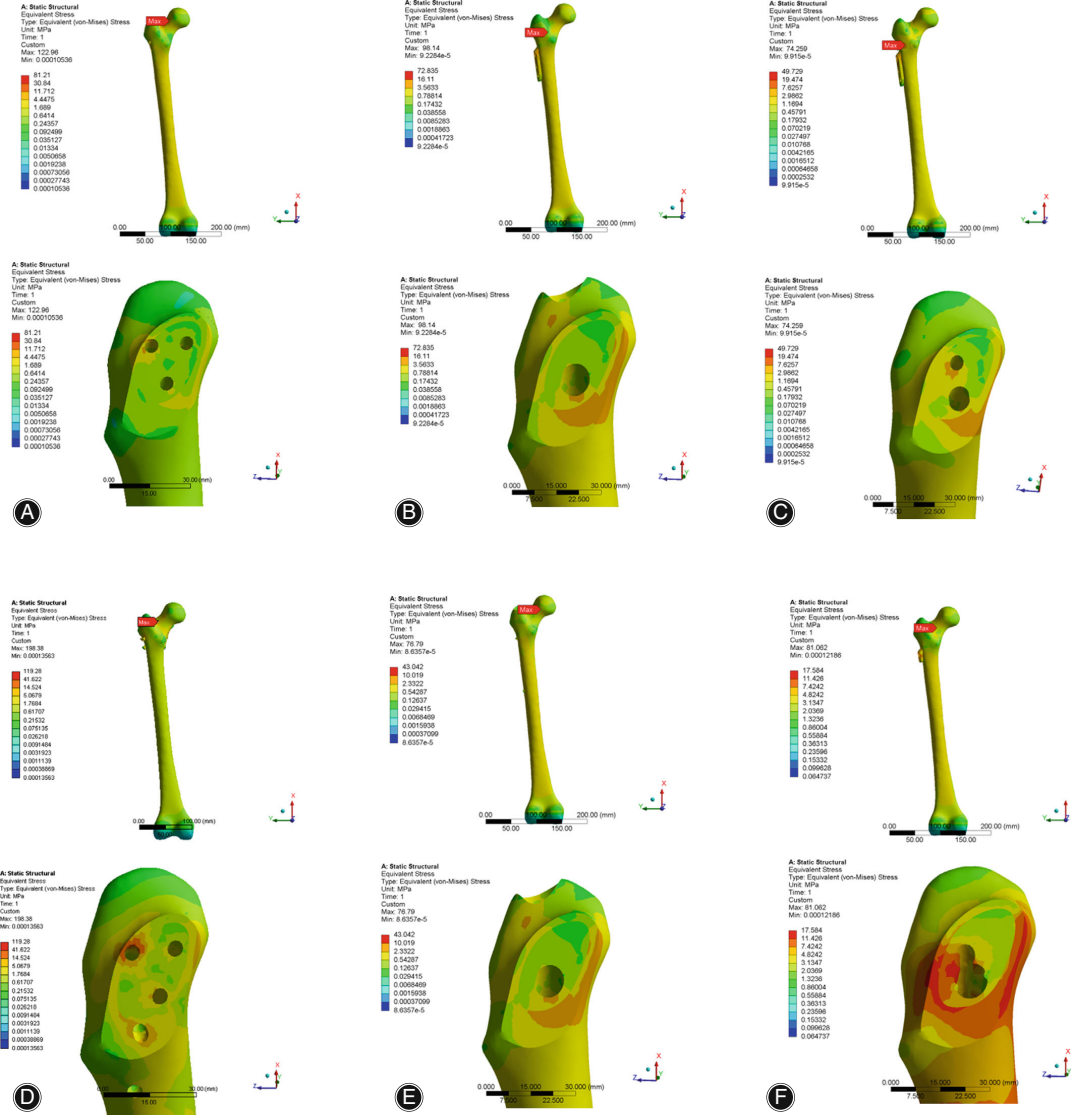
Stress distribution of femur and femur section under torsional loading. (A) CCS; (B) DHS; (C) DHS + DS; (D) CCS + MBP; (E) PFNA; (F) FNS. The maximum stress distribution of femur is mainly distributed on the broken end of the fracture and femoral calcar. CCS, cannulated compression screw; DHS, dynamic hip screw; DS, DHS with de‐rotational screw; FNS, femoral neck system; MBP, CCS with medial buttress plate; PFNA, proximal femoral nail anti‐rotation.

Under torsional loading, the stress distribution in the femur was similarly concentrated on the anterior and inferior fracture surfaces near the femoral callus. Additionally, the stress distribution in the femur was partially concentrated on the midsection of the femur shaft in the DHS, DHS + DS, and PFNA (Figure 6). The peak value of femur stress in CCS, DHS DHS + DS, CCS + MBP, PFNA and FNS were 81.2, 72.8, 74.3, 119.3, 43.0 and 17.6 MPa, respectively (Table 3, Figure S1). The CCS + MBP had maximal peak stress of femur and the FNS had the least stress.

### Displacement Distribution and Peak Value of Internal Fixation

**FIGURE 7:**
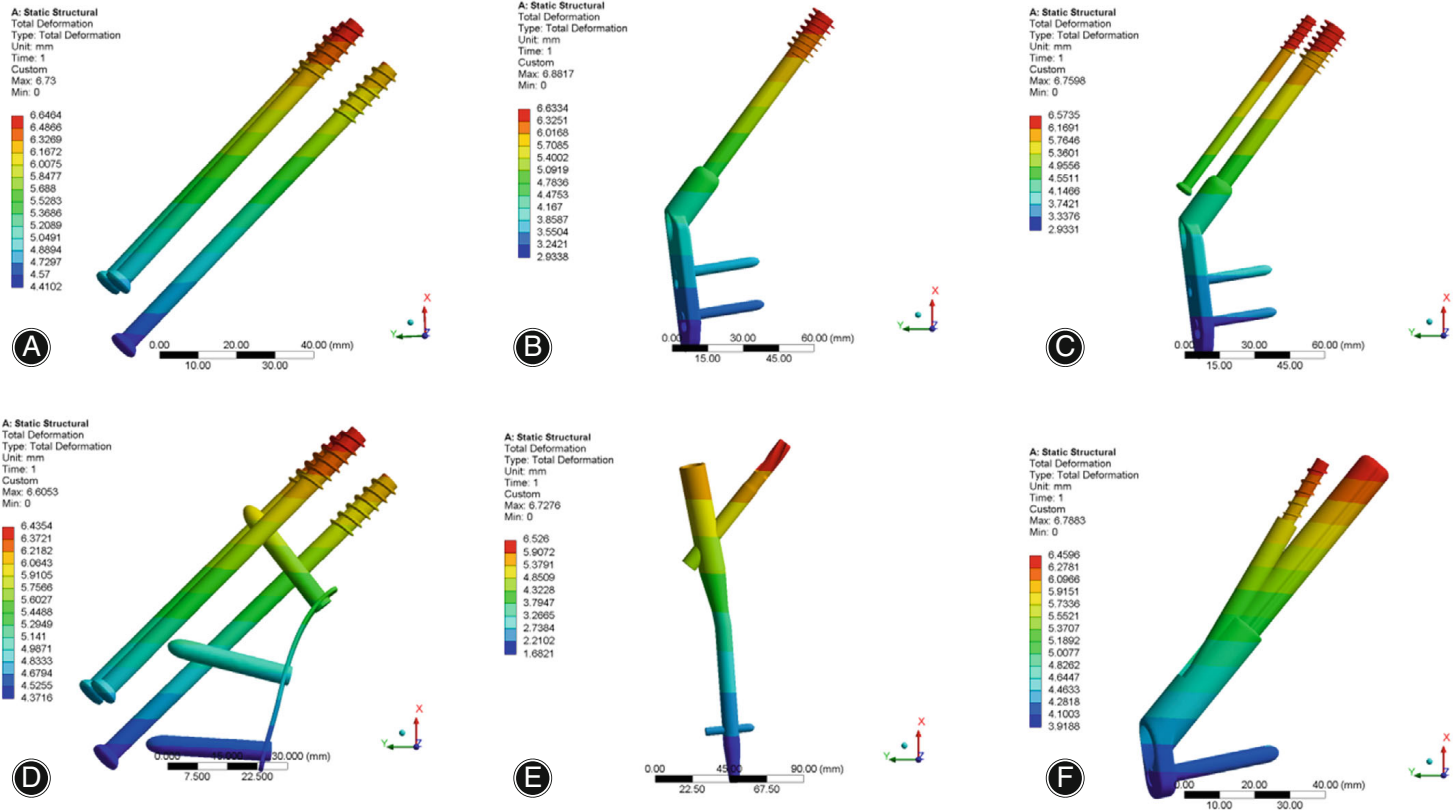
Displacement distribution in internal fixation of finite element models under axial loading. (A) CCS; (B) DHS; (C) DHS + DS; (D) CCS + MBP; (E) PFNA; (F) FNS. The displacement was mainly focused at the proximal end of the internal fixation device, and the greatest displacements were observed at the top of the various implants with a consistent distribution. CCS, cannulated compression screw; DHS, dynamic hip screw; DS, DHS with de‐rotational screw; FNS, femoral neck system; MBP, CCS with medial buttress plate; PFNA, proximal femoral nail anti‐rotation.

As shown in Figure 7, the displacement was mainly focused at the proximal end of the internal fixation device, and the greatest displacements were observed at the top of the various implants with a consistent distribution. The peak value of displacement in CCS, DHS, DHS + DS, CCS + MBP, PFNA and FNS were 6.65, 6.63, 6.57, 6.44, 6.53 and 6.46 mm, respectively (Table 4, Figure S2). The peak displacement of the CCS was the largest, and the peak displacement of the CCS + MBP was the smallest.

**TABLE 4:** Peak femoral and internal fixation displacement in six internal fixation models under different loads.

Model	Axial compression load (mm)		Horizontal torsional load (mm)	
	Femur	Internal fixation	Femur	Internal fixation
CCS	6.73	6.65	0.296	0.236
DHS	6.88	6.63	0.440	0.241
DHS + DS	6.75	6.57	0.324	0.213
CCS + MBP	6.61	6.44	0.292	0.249
PFNA	6.73	6.53	0.365	0.205
FNS	6.79	6.46	0.337	0.210

Abbreviations: CCS, cannulated compression screw; DHS, dynamic hip screw; DS, DHS with de‐rotational screw; FNS, femoral neck system; MBP, CCS with medial buttress plate; PFNA, proximal femoral nail anti‐rotation.

**FIGURE 8:**
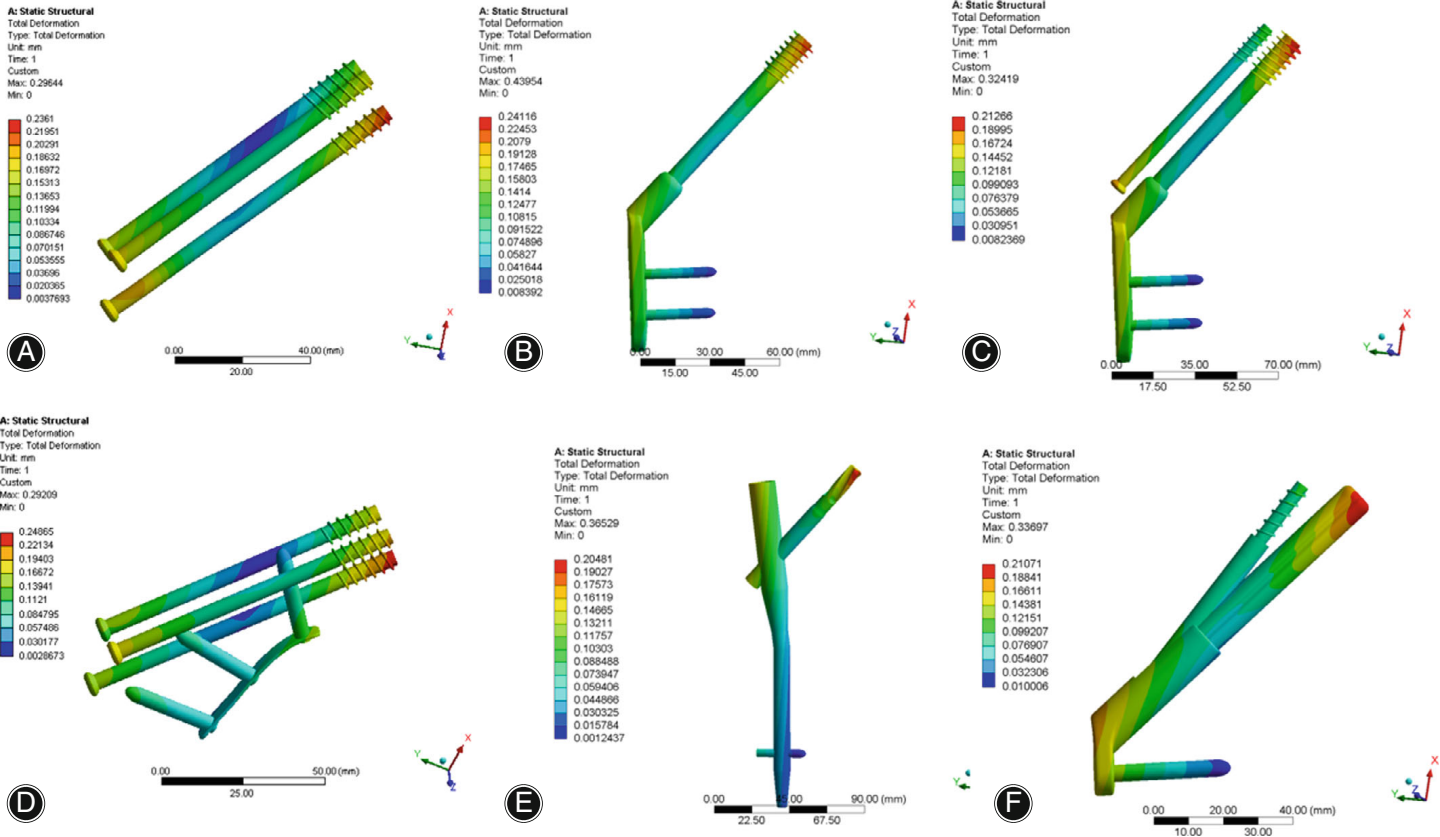
Displacement distribution in internal fixation of finite element models under torsional loading. (A) CCS; (B) DHS; (C) DHS + DS; (D) CCS + MBP; (E) PFNA; (F) FNS. The predominant focus of displacement is focused at both the proximal and distal ends of the internal fixation device. CCS, cannulated compression screw; DHS, dynamic hip screw; DS, DHS with de‐rotational screw; FNS, femoral neck system; MBP, CCS with medial buttress plate; PFNA, proximal femoral nail anti‐rotation.

Under torsional loading, the predominant focus of displacement was observed at both the proximal and distal ends of the internal fixation device. However, the highest displacements of six internal fixations were found at the top of the internal fixation. Further, in CCS, CCS + MBP, DHS + DS and FNS, the maximum displacements were occurred at top of inferior screw, lag or bolt (Figure 8). The peak value of displacement in CCS, DHS, DHS + DS, CCS + MBP, PFNA and FNS were 0.236, 0.241, 0.213, 0.249, 0.205 and 0.210 mm, respectively (Table 4, Figure S2). The peak displacement of the CCS + MBP was the largest, and the peak displacement of the PFNA was the smallest.

### Displacement Distribution and Peak Value of Femur

**FIGURE 9:**
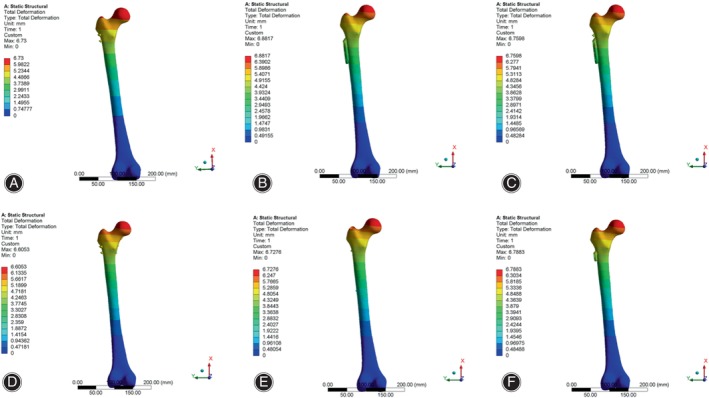
Displacement distribution in femur of finite element models under axial loading. (A) CCS; (B) DHS; (C) DHS + DS; (D) CCS + MBP; (E) PFNA; (F) FNS. The greatest femoral displacement is mainly disturbed at the upper region of the femoral head. CCS, cannulated compression screw; DHS, dynamic hip screw; DS, DHS with de‐rotational screw; FNS, femoral neck system; MBP, CCS with medial buttress plate; PFNA, proximal femoral nail anti‐rotation.

As illustrated in Figure 9, the greatest femoral displacement was observed predominantly at the upper region of the femoral head, and the peak value of femoral displacement in CCS, DHS, DHS + DS CCS + MBP, PFNA and FNS were 6.73, 6.88, 6.75, 6.61, 6.73 and 6.79 mm, respectively (Table 4, Figure S2).

**FIGURE 10:**
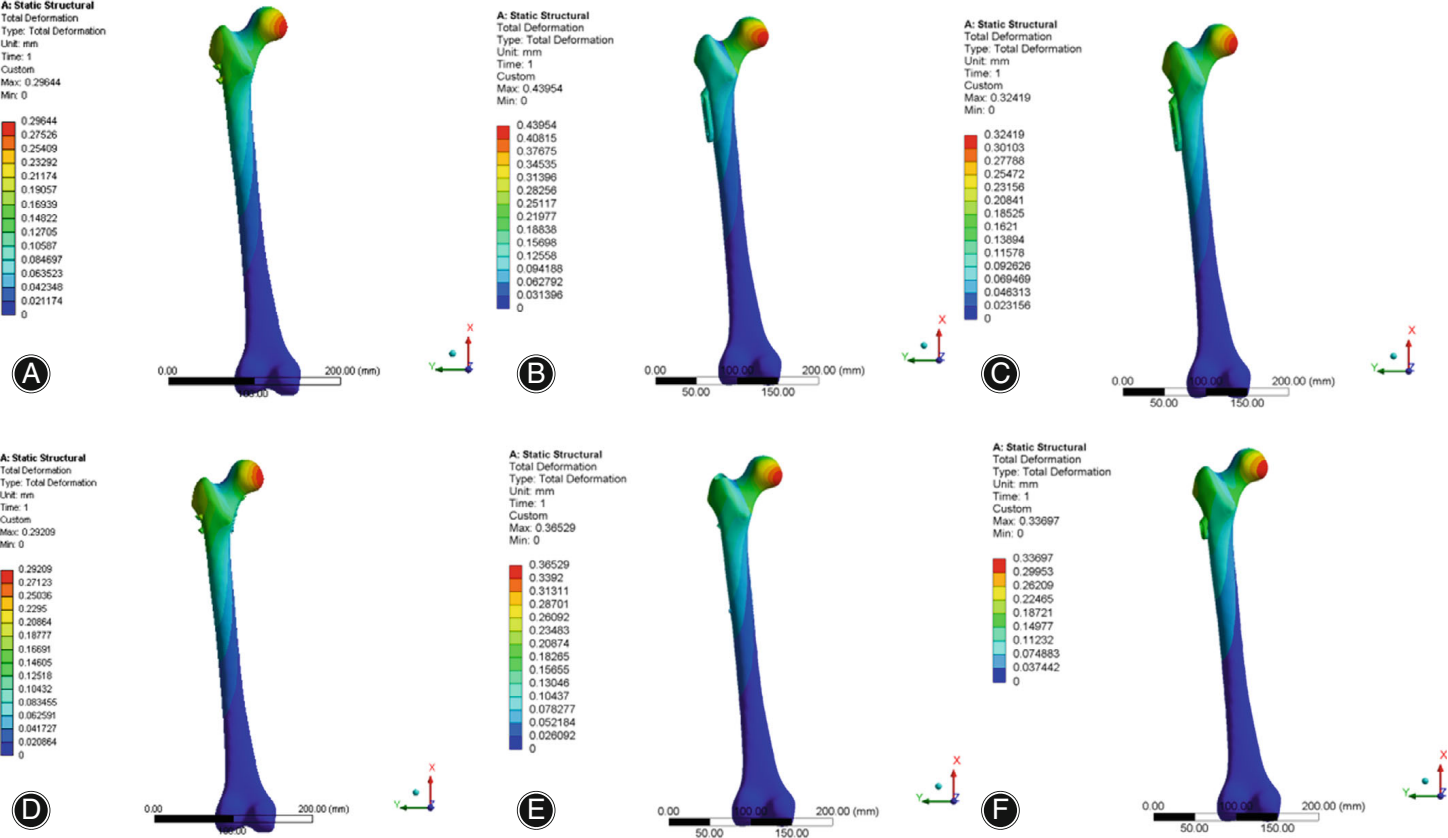
Displacement distribution in femur of finite element models under torsional loading. (A) CCS; (B) DHS; (C) DHS + DS; (D) CCS + MBP; (E) PFNA; (F) FNS. The largest femoral displacement is mainly disturbed concentrated at the lower region of the femoral head. CCS, cannulated compression screw; DHS, dynamic hip screw; DS, DHS with de‐rotational screw; FNS, femoral neck system; MBP, CCS with medial buttress plate; PFNA, proximal femoral nail anti‐rotation.

Under torsional loading, the maximum displacement of femur was concentrated at the lower region of the femoral head (Figure 10), and the peak value of femoral displacement in CCS, DHS, DHS + DS, CCS + MBP, PFNA and FNS were 0.296, 0.29, 0.440, 0.324, 0.365 and 0.337 mm, respectively (Table 4, Figure S2). Under two different conditions, the CCS + MBP had minimum peak displacement of femur and DHS had the maximal peak displacement.

### Yield Loads and Fatigue Life for Internal Fixation Model

The yield loading for the CCS, DHS, DHS + DS, CCS + MBP, PFNA and FNS were 2513, 3488, 2380, 2576, 2600 and 3071 N, respectively (Table 5, Figure S3). The fatigue lives of the CCS model, DHS model, DHS + DS, CCS + MBP, PFNA and FNS were 7.01 × 10^7^ cycles, 1.15 × 10^7^ cycles, 5.91 × 10^7^ cycles, 9.41 × 10^7^ cycles, 7.49 × 10^7^ cycles and 7.43 × 10^7^ cycles (Table 5, Figure S4). All six internal fixation models showed excellent performance in terms of yield load and fatigue life.

**TABLE 5:** Axial compressive yield load and fatigue life of a six‐group internal fixation model.

Model	compressive yield load (N)	Fatigue life (×10^7^)
CCS	2513	7.01
DHS	2380	5.91
DHS + DS	2576	9.41
CCS + MBP	3488	1.15
PFNA	2600	7.49
FNS	3071	7.43

Abbreviations: CCS, cannulated compression screw; DHS, dynamic hip screw; DS, DHS with de‐rotational screw; FNS, femoral neck system; MBP, CCS with medial buttress plate; PFNA, proximal femoral nail anti‐rotation.

## Discussion

In the current investigation, a comprehensive examination was conducted to analyze the biomechanical implications of six internal fixation methods for addressing Pauwels type III femoral neck fractures through finite element analysis. The findings showed that CCS + MBP exhibited superior initial mechanical stability, albeit with increased vulnerability to shear stress. Conversely, DHS + DS and FNS were identified as effective fixation techniques conducive to fracture healing. Due to the significant shear force and tension at the fractured end, there was a high frequency of internal fixation failure, postoperative complications, and the need for reoperation in patients with Pauwels III type femoral neck fractures.^24^ Consequently, numerous research efforts have been directed toward developing an efficient internal fixation strategy capable of addressing the unstable biomechanical conditions and ensuring long‐term mechanical stability in Pauwels type III fractures.^25^ Up to this point, because of lacking substantial evidence, there has been significant debate regarding the selection of the most effective internal fixation device for managing Pauwels type III type fractures.^12^ Due to constraints on participant enrollment, variations in fixation methods, and differing test conditions in biomechanical and clinical studies, this study utilized finite element analysis to systematically examine the biomechanical properties of six prevalent internal fixation methods for treating Pauwels III type fractures. Several significant findings from our study could be delineated as follows: (i) the stress distribution was primarily concentrated in the middle of the internal fixation device near the fracture site, with slight variations depending on their configuration under different loadings; (ii) it was evident that the peak stress in the fixation modalities was significantly higher than that in the femur, indicating that internal fixation effectively bore most of the stress, which was beneficial for the early stage of healing; (iii) the CCS + MBP model exhibited the highest peak stress on both the internal fixation and femur under both axial and torsional loading, while the CCS model had the lowest peak stress on both the internal fixation and femur under axial loading, however the DHS + DS and FNS models showed the lowest peak stress on the internal fixation and femur, respectively, under torsional loading; (iv) the CS + MBP model demonstrated the least displacement of the femoral head under both axial and torsional loading, while the DHS model had the maximum displacement; and (v) in the yield test and fatigue test, all six internal fixation models displayed excellent mechanical stability, additionally, the CS + MBP model exhibited the highest yield loading and the lowest number of cyclic loading.

### Biomechanical Outcomes of Six Devices in Treatment of Pauwels Type III Fractures

#### CCS

To date, the fixation methods of CCS and DHS have remained the most prevalent and favored surgical approaches for addressing Pauwels type III femoral neck fractures.^9^ CCS offered several advantages, including reduced soft tissue damage, maximal preservation of femoral head blood supply, decreased blood loss, shorter surgical duration, and reduced hospitalization period for patients.^24^, ^26^, ^27^ Our study showed that, the “inverted triangle” CCS model had weak ability against vertical shear but had high torsional resistance in fixation of Pauwels type III fractures. The rationale for this phenomenon might be attributed to the augmented cross‐sectional area and the structurally robust triangular arrangement of multiple screws, which possess the capability to distribute stress from the upper part of the femoral head to the calcar, making it more conducive for weight bearing.^15^, ^28^ Nevertheless, similar to our investigation, previous finite element analyses had suggested that CCS exhibited inadequate mechanical stability in withstanding high shear and inversion forces at fracture sites, potentially leading to varus deformity, nonunion, femoral neck shortening and femoral head necrosis after surgery.^17^, ^29^ Likewise, Liporace *et al*.^4^ showed that the overall rate of postoperative complications was 32% in the fixation of Pauwels type III fractures using an “inverted triangle” CCS, with a non‐union rate of 19% and an incidence of femoral head necrosis rate of 14%. Consequently, based on our findings, we did not advocate for the use of CCS in the fixation of Pauwels type III fractures. In comparison to CCS, several biomechanical studies have confirmed that DHS exhibits superior shear resistance, fixation strength, and angular stability, although it has a weaker ability to counter rotation when used alone.^30^, ^31^

#### DHS

Numerous studies have suggested the incorporation of a DS with DHS fixation, as this combination has the potential to enhance anti‐rotation capabilities and reinforce the compressive effect on the fractured end, aiding improved axial and rotational stability and a reduced incidence of internal fixation failure.^32^ Ma *et al*.^33^ and Freitas *et al*.^34^ analyzed the biomechanical performance of three internal fixation models (CCS, DHS and DHS + DS), and showed that the DHS + DS model demonstrated superior resistance to rotation and shearing, making it a recommended choice for Pauwels type III femoral neck fractures. Our study also found that the maximum displacement and stress experienced by the internal fixation and femur in the DHS + DS were both reduced compared to the DHS, and anti‐rotation performance of the DHS + DS closely resembled that of the CCS. However, it was important to note that the addition of a DS resulted in longer operative times, increased intraoperative bleeding, and greater damage to bone and blood supply, which might ultimately elevate the risk of femoral head necrosis following surgery.^35^ Moreover, Zhang *et al*.^36^ demonstrated a notable prevalence of femoral head necrosis and femoral neck shortening in Pauwels type III fractures treated with DHS + DS fixation, which was ascribed to the persistent compressive characteristics of DHS, leading to heightened bone resorption at the fracture site and impeding excessive femoral blood flow. In addition, even though there is widespread adoption of DHS + DS, this approach might result in not only damage to the adductor muscles but also varying levels of abduction pain.^37^

#### CCS + MBP

Generally speaking, in finite element model, the displacement of the femoral head was indicative of the internal fixation's capacity to uphold stability at the fracture site and withstand the inversion and shortening of femoral neck.^17^ MBP, was specifically designed to address the substantial shear forces associated with Pauwels type III fractures, transforming these forces at the fracture site into stress forces that not only facilitated fracture healing but also exhibited excellent anti‐slip and anti‐rotation properties.^38^ Our study found that the CCS + MBP model had an optimally initial mechanical stability when compared to other five internal fixations. Previous biomechanical and clinical studies had suggested that, in contrast to the use of CCS alone, CCS + MBP maintained sliding compression and anti‐rotation capabilities, while also directly resisting shear stress, contributing to a more favorable prognosis for the treatment of Pauwels type III fractures.^39^, ^40^ However, because of intricate anatomical positioning, the placement of MBP medially of the femoral neck posed significant challenges, potentially leading to joint capsule damage or hip impingement.^41^, ^42^ Moreover, there was a potential risk of compromising the blood supply to the femoral head during the surgical procedure, particularly the inferior supporting artery, which is crucial for post‐operative revascularization and fracture healing.^41^, ^42^ Similar to a prior study,^43^ our results indicated that the MBP exhibited both maximal peak stress and the minimum number of cycle loads, potentially increasing the risk of implant breakage. Despite the plate breakage rate was low in clinical practice, further investigations should be conducted to verify the mechanical reliability and internal fixation‐related complications associated with CCS + MBP treatment.^44^

#### PFNA

Previous studies have indicated that, PFNA had the capacity to provide extended intramedullary support, effectively transmitting and dispersing shear forces from the fracture site to the cortex of the femoral shaft, affording sufficient biomechanical strength for resisting shear forces and structural displacement.^45^ Concurrently, some evidence suggest that PFNA demonstrated superior ability to prevent anti‐femoral neck shortening and decreasing risk of implant failure when confronted with huge vertical shearing in Pauwels type III fractures.^45^, ^46^ In this study, it was observed that the PFNA exhibited favorable mechanical properties, characterized by low peak stress and displacement of the femoral head. However, when compared to five other internal fixation devices, the biomechanical stability advantage was not readily apparent. Furthermore, the use of intramedullary fixation for femoral neck fractures posed a high risk of reverse wedge effect, potentially leading to loss of compression at the fracture site and mechanical cut‐out failure. Additionally, this method was associated with greater interference with the proximal femoral cavity, tissue damage, and concealed blood loss.^47^ Consequently, the application of PFNA for Pauwels type III fractures was infrequent in clinical practice.

#### FNS

FNS was a recently developed form of internal fixation intended to combine the advantages of various existing implants, with the theoretical mechanical advantage of combining compression and anti‐rotation during internal fixation.^48^ Our study demonstrated that, the FNS model showed the second smallest peak displacement of the femoral head, as well as the lowest peak stress on the femoral head. Additionally, in both compressive yield loading and cyclic loading fatigue tests, the FNS exhibited excellent mechanical stability and sufficient stiffness when subjected to shear loading and overturning stress from Pauwels type III fracture, which was attributed to its structural design. On the one hand, in the FNS, the bolt and anti‐rotation screw were combined to form a cohesive unit, offering robust angular support and effectively preserving the position of the fracture; on the other hand, the FNS had the capability to be secured to the femoral shaft using a distal locking screw, potentially diminishing stress and displacement on the femur, thereby establishing a favorable mechanical milieu for the healing of the fracture.^48^, ^49^ The latest evidence had indicated that the efficacy of FNS treatment surpassed that of CCS in addressing femoral neck fractures. For instance, Patel *et al*.^50^ demonstrated that FNS presented a more favorable alternative for femoral neck fractures, exhibiting lower complication rates, faster union, and improved clinical outcomes;^50^ Niemann *et al*.^51^ observed that FNS facilitated the restoration of hip geometry, including leg length, femoral offset, and collodiaphysial angle, resulting in similarly high functional and global health scores as those achieved with DHS. However, the therapeutic effect and biomechanical stability of FNS in the treatment of Pauwels type III fractures was still controversial. Ma *et al*.^33^ discovered that the maximum stresses experienced by the internal fixation and fracture end in the FNS model were greater than those in the four CCS model and DHS + DS model. Moreover, Xia *et al*.^52^ showed that the structural integrity of FNS was inferior to that of CCS, CCS + MBP, and biplanar support CCS, indicating that FNS might not possess the stability previously reported. Additionally, Jiang *et al*.^53^ proposed that FNS failed to achieve sufficient triangular support base and area moment of inertia compared to CCS, resulting in reduced resistance to axial motion. Therefore, FNS, as a newly developed internal fixation device, more biomechanical and long‐term follow‐up clinical studies need to be further carried out.

Consequently, the study only captured the force on the femur during specific movements and would benefit from being integrated with a kinetic analysis of the musculoskeletal system to yield more precise and applicable simulation results for clinical use.

### Limitations and Strengths

There were certain limitations in this study as follows: (i) the biomechanical simulation of finite element models was based on the commonly used static fixation method. In real life, the hip joint is mainly subjected to dynamic forces during activities such as walking and jumping, and the presence of soft tissues, such as muscles, also influences the force on the femur. Consequently, this study only captured the force on the femur during specific movements, and it need to be combined with kinetic analysis of the musculoskeletal system to yield more precise and applicable simulation results for clinical use; (ii) all fracture types and internal fixation placement position in the study were established from a single volunteer, making our study subject‐specific; and (iii) as a preliminary basic study, this research exclusively relied on finite element analysis techniques and does not involve any biomechanical testing using human subjects. Although the study had limitations, a notable strength of this study was that, there had been no previously systematic biomechanical research that compared these six devices for treating Pauwels III type fractures; additionally, it was the first finite element analysis to simulate various mechanical conditions specific to Pauwels III type fractures such as axial compression loading, horizontal torsion loading, yield loading and cyclic loading. Yet, on the strength of our initial findings, more studies are urgently needed for additional biomechanical experiments and clinical research to determine the most suitable internal fixation method for Pauwels III type fractures.

### Prospect of Clinical Application

Based on the findings at the biomechanical level and previous clinical investigations, the following recommendations are proposed for the clinical management of Pauwels III type fractures: (i) while the prevailing surgical techniques for unstable femoral neck fractures in young adults currently involve multiple CCS and DHS procedures, it is suggested that enhancing these internal fixation methods, such as incorporating MBP with CCS or DS with DHS, may enhance stability and facilitate fracture healing; (ii) it is important to consider that utilizing CCS + MBP could compromise the sliding compression properties of CCS, potentially leading to increased stress concentration and fracture susceptibility at the MBP site. Additionally, this surgical approach may extend operation duration, escalate surgical bleeding, and elevate healthcare expenses; (iii) FNS, a novel development, provides biomechanical stability similar to traditional internal fixation devices while also offering benefits such as decreased surgical trauma and reduced need for intraoperative fluoroscopy, which can be considered as a viable treatment option for Pauwels III femoral neck fractures.

## Conclusion

In our study, it was found that the CCS + MBP had the best initial mechanical stability in treatment for Pauwels III type fracture, and the MBP was found to be more susceptible to shear stress, thereby increasing the risk of plate breakage. Among other conventional treatment approaches, DHS + DS was superior to CCS, DHS and PFNA, providing a more stable biomechanical environment for fracture healing. In addition, it appeared that FNS was an effective fixation method for Pauwels III type fracture. However, further biomechanical experimental and clinical inquiries are warranted to elucidate our observations.

## Conflict of Interest Statement

The authors declare that they have no competing interests.

## Author Contributions

SX and HZ conceived the study and wrote the manuscript; CDD performed the finite element analysis; HC and XMQ reconstructed the models; ZXT and DQ collected and analyzed the data.

## Supporting information


**Figure S1.** Peak femoral and internal fixation stresses in six internal fixation models under different loadings.
**Figure S2.** Peak femoral and internal fixation displacement in six internal fixation models under different loadings.
**Figure S3.** Axial compressive yield load of a six‐group internal fixation models.
**Figure S4.** Fatigue life of a six‐group internal fixation models.
