# Supplementary material for: Finite Element Analysis of Six Internal Fixations in the Treatment of Pauwels Type III Femoral Neck Fracture
Source: Orthop Surg. 2024 May 15;16(7):1695–709. doi: 10.1111/os.14069 (PMC11216836; doi:10.1111/os.14069)
Supplement: Supplementary file 1 — Figure S1. Peak femoral and internal fixation stresses in six internal fixation models under different loadings. Figure S2. Peak femoral and internal fixation displacement in six internal fixation models under different loadings. Figure S3. Axial compressive yield load of a six‐group internal fixation models. Figure S4. Fatigue life of a six‐group internal fixation models. [file OS-16-1695-s001.docx]

**Supplementary figures**

**
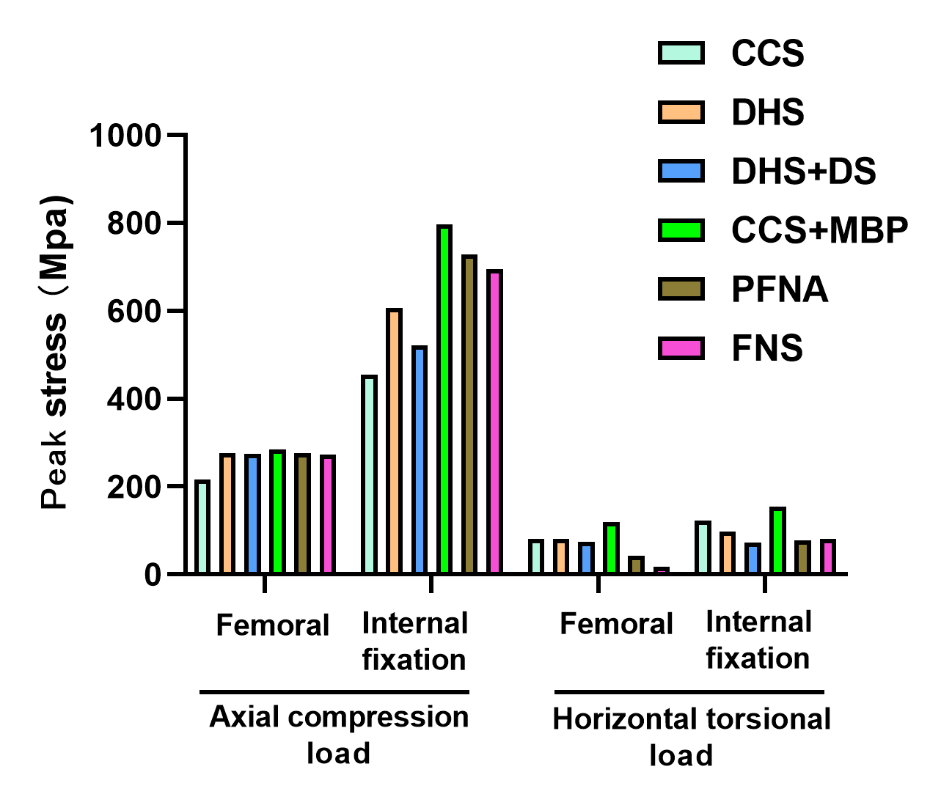
**

**Supplementary figure1.Peak femoral and internal fixation stresses in six internal fixation models under different loadings.**

**
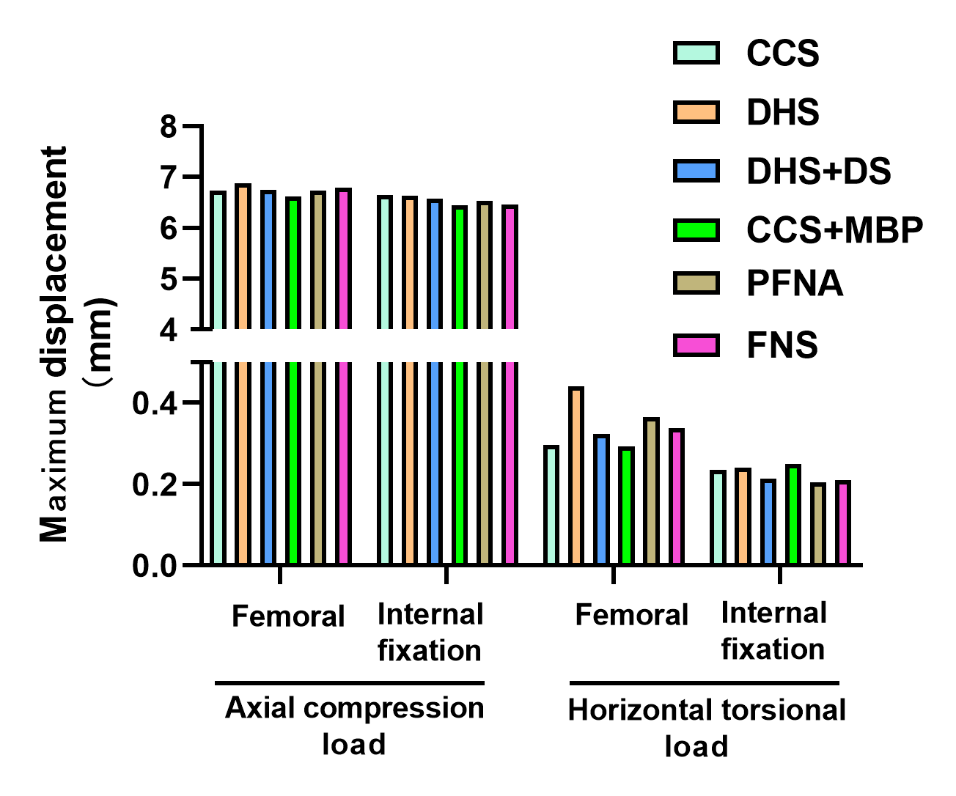
**

**Supplementary figure2. Peak femoral and internal fixation displacement in six internal fixation models under different loadings.**

**
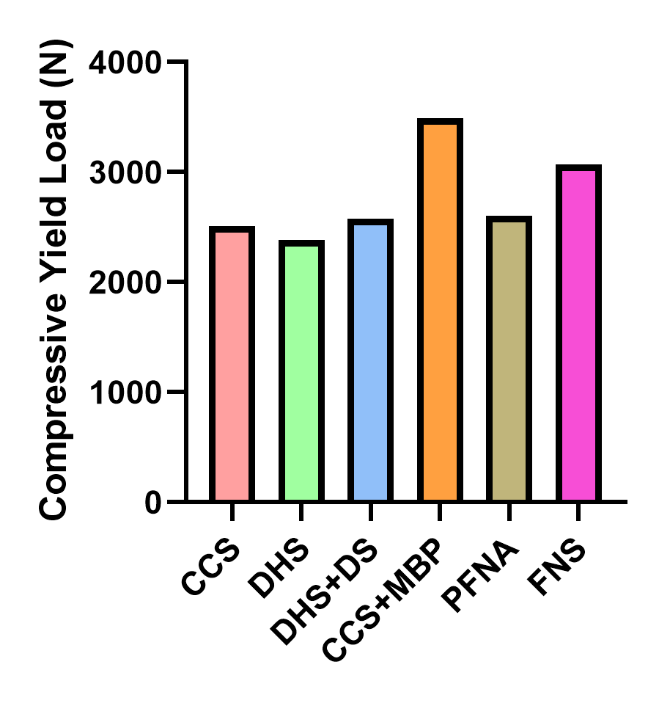
**

**Supplementary figure 3. Axial compressive yield load of a six-group internal fixation models**

**
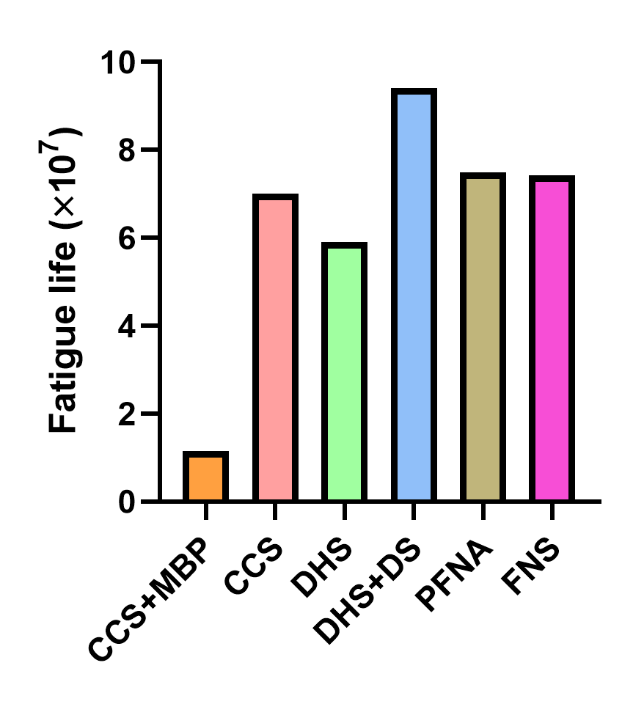
**

**Supplementary figure 4. Fatigue life of a six-group internal fixation models**
